# Evolutionary History and Ecology of *Andrena* (*Foveoandrena*) *androfovea*: A New Nearctic Mining Bee (Hymenoptera, Andrenidae) Species and Subgenus

**DOI:** 10.1002/ece3.70453

**Published:** 2024-11-03

**Authors:** Silas Bossert, Keng‐Lou James Hung, John L. Neff

**Affiliations:** ^1^ Department of Entomology Washington State University Pullman Washington USA; ^2^ Department of Entomology National Museum of Natural History, Smithsonian Institution Washington DC USA; ^3^ Oklahoma Biological Survey University of Oklahoma Norman Oklahoma USA; ^4^ Central Texas Melittological Institute Austin Texas USA

**Keywords:** *Chamaesaracha*, divergence times, pollen‐collecting, *Quincula*, solitary bees, ultraconserved elements

## Abstract

With about 1700 described species, the mining bee genus *Andrena* is a rapidly diversifying lineage and one of the most species‐rich groups of bees. Recent phylogenomic advances have greatly improved our understanding of the phylogeny of the genus, yet many species still await description, subgeneric assignments that are in line with their evolutionary history, as well as study of their morphology and behavior. Here we provide a comprehensive account of a newly discovered species, *Andrena androfovea* n. sp. We sequence the genome of the new species and include it in the presently most comprehensive phylogenomic analysis of *Andrena* using ultraconserved element (UCE) sequence data, comprising 264 samples and 249 species. Given the recovered phylogenetic position of the new species, we establish a new subgenus, *Foveoandrena*, provide a detailed morphological description, and discuss the antiquity and historical biogeography of the lineage in light of molecular divergence time estimates. Lastly, we study and document the foraging behavior of the new species with photos and video footage, and discuss the species' unusual host plant associations with *Chamaesaracha* and *Quincula*, both Solanaceae. Being likely oligolectic on these plants, we present the first documented case of an *Andrena* species being narrowly associated with members of this plant family. By integrating multiple lines of documentation, our study provides a particularly detailed account of species discovery and description.

## Graphical abstract

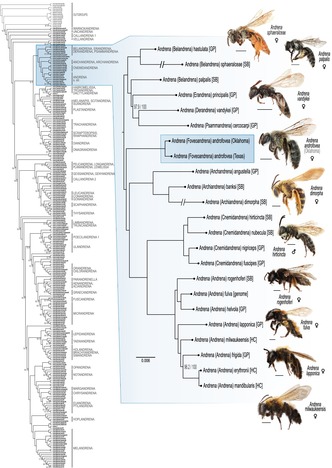

## 1 Introduction

Approximately 8% of global bee species are members of the mining bee genus *Andrena* Fabricius, 1775 (Hymenoptera, Andrenidae), a group of taxonomically, biologically, morphologically, and behaviorally diverse pollinating insects. *Andrena* are primarily solitary bees; each individual female constructs and provisions her own nest cells in the soil. While many species of *Andrena* nest in large aggregations of hundreds or even thousands of individual nests (Bischoff 2003; e.g., Friese 1882; Michener and Rettenmeyer 1956; Neff and Simpson 1997), and others nest communally with multiple females sharing a common nest entrance (Paxton and Tengö 1996), none are truly social as each female forages independently for nectar and pollen, and provisions and lays eggs in her own brood cells. Species of *Andrena* have long been studied for their pollen‐collecting behavior, as the genus includes a heterogeneous mix of pollen specialists (oligoleges) that collect pollen from a single plant family, genus, or even rarely, species, and species with broader diets of pollens from a variety of host plant families (polyleges; Danforth, Minckley, and Neff 2019; Dermane et al. 2021; Larkin, Neff, and Simpson 2006; Robertson 1925; Wood and Roberts 2018).

With approximately 1700 currently described species, *Andrena* is monophyletic, very species‐rich, and one of the most rapidly diversifying lineages across all bees (Bossert et al. 2022). To this day, *Andrena* taxonomy is an active field of research, and no other genus of bees saw a greater number of newly described species in the present decade (i.e., from 2020 to the present day). Historically, this diverse taxon has been classified into a large number of subgenera, with the more recent classifications recognizing about 100 subgenera: Gusenleitner and Schwarz (2002) list 99 subgenera, Ascher (2004) lists 95, Michener (2007) lists 96, and Dubitzky, Plant, and Schönitzer (2010) lists 101. The differences in the subgeneric classifications, the frequently unclear morphological delineation between subgenera, and the inaccessibility of rare or dubious species for study, either because of rarity in the field or unavailable type specimens, have presented persistent, long‐standing problems for *Andrena* taxonomists. Specifically, it often proved very challenging to associate undescribed species of *Andrena* with a described subgenus or assess the affinity to other, already described species. This situation recently changed. Systematic‐taxonomic research on *Andrena* gained significant momentum with the comprehensive, molecular phylogeny‐based assessment of *Andrena* subgeneric relationships by Pisanty, Richter, et al. (2022). In that paper, Pisanty, Richter, et al. (2022) sampled over 200 species of *Andrena* from 98 nominal subgenera, providing the first comprehensive analysis that integrates both molecular and morphological information at a large scale. This information provided a new taxonomic framework for about 100 newly described species of *Andrena* since 2022 (Neff 2024; Pisanty et al. 2023; Pisanty, Scheuchl, et al. 2022; Wood 2021, 2022, 2023a, 2023b, 2023c, 2024; Wood et al. 2021, 2023, 2024; Wood and Monfared 2022), some of which were specifically included in their phylogeny (e.g., *Andrena nahua* Neff, *Andrena inusitata* Pisanty). Additionally, new subgenera have been described in light of the new molecular evidence (Wood and Pisanty 2024). However, the phylogenetic‐taxonomic status of many species of *Andrena* still remains uncertain, and even newly described species are frequently treated as ‘incertae sedis’ (e.g., Pisanty, Scheuchl, et al. 2022; Wood 2023b; Wood and Pisanty 2024). Shortly following the publication of Pisanty, Richter, et al. (2022), Bossert et al. (2022) published a densely sampled phylogenomic analysis of Andrenidae as a whole, including 74 species of *Andrena* in 36 subgenera. While some species are shared between Pisanty, Richter, et al. (2022) and Bossert et al. (2022), about one‐third of Bossert et al.'s (2022) *Andrena* are not included in Pisanty, Richter, et al.'s (2022) phylogeny.

In the present paper, we expand our understanding of *Andrena* phylogeny, taxonomy, and natural history with a fourfold approach. (1) First, we expand the phylogenetic framework of Pisanty, Richter, et al. (2022) by combining their *Andrena* sequence data with those of Bossert et al. (2022). (2) We bioinformatically optimize the recovery of genomic ultraconserved elements (UCEs) by comparing different UCE contiguous sequence (contig) assemblies and include sequence data from the currently available *Andrena* genomes. (3) We sequence the genome of a previously undescribed Nearctic species of *Andrena*, embed it into the newly estimated phylogeny, and evaluate its subgeneric relationships. We then describe, diagnose, and illustrate the new species, *Andrena* (*Foveoandrena*) *androfovea* n. sp. and n. subg., and place it into a newly described subgenus. (4) Lastly, we provide information on the biology of the new species and document its foraging behavior.

## 2 Material and Methods

As a principal step for studying newly described *Andrena*, we developed an improved and expanded phylogenetic framework for the genus. To this end, we combined sequence data from Pisanty, Richter, et al. (2022) and Bossert et al. (2022), included sequence data from eight publicly available genomes, as well as sequences from four additional *Andrena* genomes that were generated in the present study.

### 2.1 Acquisition of New Whole Genome Data

DNA was extracted from the tissue of three legs for each specimen. We used the Zymo Quick‐DNA Miniprep Plus Kit (Zymo Research Corp.) according to the manufacturer's protocol, except that we eluted the DNA off the spin column with 120 μL of elution buffer. Voucher specimens that correspond to the DNA extracts are deposited in the research collection of the first author, which is housed in the Washington State University Insect Collection (WSUC). Voucher specimens have been associated with a voucher label and the following voucher codes: WSUX_AV4 for a representative of *Andrena androfovea* n. sp. from the collecting site in Texas (see collection details below), WSUX0070 for a specimen of *Andrena androfovea* n. sp. from Oklahoma, WSUX_AV1 for *Andrena* sp. ‘C’, and WSUX_AV2 for *Andrena* sp. ‘L’. After DNA extraction, we quantified double‐stranded DNA with a Qubit 3.0 fluormeter (Thermo Fisher Scientific, Inc.) and assessed fragmentation by running 2 μL of each sample on a high‐sensitivity screening tape in a 4150 TapeStation System (Agilent Technologies, Inc.). We sheared a total of 50 ng of each sample to an approximate target fragment size of 300–500 bp with a BioRuptor 300 (Diagenode, Inc.). Following sonication, we prepared sequencing libraries for Illumina platforms using a KAPA HyperPrep Kit (F. Hoffmann‐La Roche, Ltd.). We followed the protocol outlined in Branstetter et al. (2021), which starts with a purification step of the input DNA using magnetic beads at a 3:1 ratio (3.0× SPRI). The libraries were dual‐indexed (Glenn et al. 2019) and we used the 2× TaKaRa Ex Premier DNA Polymerase mastermix (Takara Bio USA, Inc.) instead of the KAPA kit's PCR mastermix. We ran a total of 15 PCR cycles, pooled the samples at equimolar concentrations and size selected the pool to a fragment distribution of 250–650 bp with a BluePippin device (Sage Science, Inc.). Sequencing was carried out on an Illumina NovaSeq X Plus sequencer and a 10B flow cell by Novogene Co. Ltd.

### 2.2 Bioinformatics Processing of New Genomes

The bioinformatic processing of the newly generated whole‐genome data follows the steps detailed in Bossert et al. (in revision), including identical use of parameters. Briefly, this workflow comprises demultiplexing of raw Illumina reads using *demuxbyname*, which is part of BBtools v. 39.06 (https://sourceforge.net/projects/bbmap/), duplicate read removal and reordering using *Clumpify* (BBtools), adapter trimming with *fastp* v. 0.23.4 (Chen et al. 2018), and read normalization to a target coverage of 20× (minimum 2×) with *BBnorm* (BBtools). These cleaned, reordered reads were then assembled using SPAdes v. 3.15.4 (Bankevich et al. 2012) and a coverage cutoff of 5.

### 2.3 Phylogenomic Framework of *Andrena*

We obtained the raw UCE sequence data from the comprehensive phylogenetic study of Pisanty, Richter, et al. (2022), as well as their assembly files, which were generated with the short‐read assembler ABySS v. 1.3.7 (Simpson et al. 2009). Recent comparison showed significant variation in UCE recovery when using different assemblers (Bossert et al. 2024), with SPAdes outperforming other de‐novo assemblers in most, but not all cases. To optimize UCE recovery, we re‐assembled the raw sequence data of Pisanty, Richter, et al. (2022) with SPAdes, and then compared UCE recovery from both ABySS and SPAdes assemblies. To this end, we ran the Phyluce ver. 1.7.2 (Faircloth 2016) script phyluce_assembly_match_contigs_to_probes (with ‐‐min_coverage 80 and ‐‐min_identity 80) against the HymV2 principal bait set (Branstetter et al. 2017), compared the number of captured UCE loci for every *Andrena* and *Cubiandrena* species using both assemblers and only retained those assembly files that led to the greater recovery of UCEs. Following this comparison, we included all *Andrena* samples from Bossert et al. (2022), all andrenine samples outside of *Andrena* (i.e., *Alocandrena*, *Ancylandrena*, *Megandrena*, *Orphana*, *Euherbstia*), as well as two well‐performing outgroup samples representing the andrenid subfamilies Panurginae and Oxaeinae. In case identical species were sampled by both Pisanty, Richter, et al. (2022) and Bossert et al. (2022), we compared UCE recovery and discarded the sample which had lower UCE capture success. For including UCE sequences from *Andrena* whole genome data, we downloaded the eight publicly available *Andrena* genomes that were available on NCBI as of January 2024 with the following assembly identifiers: *Andrena camellia—*GCA_029448645.1, *Andrena haemorrhoa*—GCA_910592295.1, *Andrena dorsata*—GCA_929108735.1, *Andrena minutula*—GCA_929113495.1, *Andrena hattorfiana*—GCA_944738655.2, *Andrena fulva*—GCA_946251845.1, *Andrena bucephala*—GCA_947577245.1, and *Andrena trimmerana*—GCA_951215215.1. We extracted UCEs from the genomes using the Phyluce pipeline, requiring an 80% overlap and 80% identity of UCEs (see Bossert and Danforth (2018) for discussion on these parameters) to corresponding bait sequences of the ‘bee‐ant‐specific hym‐v2 bait set’ (Grab et al. 2019). For the four newly sequenced genomes, we used slightly lower overlap (70) and identity (75) thresholds. This amounted to a total of 264 analyzed samples, including the four newly sequenced samples as described below. After UCE identification, we extracted sequences with 1000 bp of flanking DNA (‐‐flanks 1000) and treated them as UCE contigs for further processing in Phyluce. To this end, we aligned UCE data using MAFFT (Katoh and Standley 2013) and trimmed poorly aligned positions with Gblocks (Castresana 2000). Following the assessment of different percentages of matrix completeness, we finalized an 80% completeness matrix, meaning that the sequence matrix includes all UCEs that were recovered by at least 80% of all included samples (=211 samples). This led to a sequence matrix comprising 1175 individual UCE loci.

### 2.4 Molecular Phylogeny

Using the UCE sequence matrix, which consists of 1175 concatenated individual UCE loci, we reconstructed the phylogeny of *Andrena* using the Maximum Likelihood (ML) software IQ‐Tree2 v. 2.3.1 (Minh et al. 2020). For partitioning the data, we used the software CURE v. 1.0.5 (Freitas et al. 2023) and their implementation of the sliding‐window site characteristics (SWSC) partitioning for UCEs, which was originally described by Tagliacollo and Lanfear (2018). Following the identification and partitioning of UCE core and left and right flanking regions, we used ModelFinder (Kalyaanamoorthy et al. 2017) to assess the best‐fitting partitioning scheme for merged partitions (iqtree2 ‐m MFP+MERGE). Here we used a threshold of 20% for the clustering algorithm (originally described by Lanfear et al. 2014) to reduce the computational time (rcluster 20). Subsequently, we used IQ‐Tree2 to reconstruct the phylogeny using the 196 designated partitions with their respective best‐fitting substitution models. Node support was assessed with 1000 SH‐like approximate likelihood ratio tests (SH‐aLRT; Guindon et al. 2010) and 1000 ultrafast bootstrap approximations (UFBoot2; Hoang et al. 2018).

### 2.5 Divergence Time Estimates

To understand the temporal origin of the newly described species, we approximated divergence times of *Andrena* using RelTime (Tamura et al. 2012), which is part of the MEGA 11 package (Tamura, Stecher, and Kumar 2021). Thorough Bayesian divergence dating has been carried out by all three recent studies on andrenid phylogeny (Bossert et al. 2022; Pisanty, Richter, et al. 2022; Ramos, Martins, and Melo 2022) and their estimates largely overlap. Hence, we employed a set of eight secondary calibrations, which we used to inform divergences of the early Andreninae, as well as splits within *Andrena* (Table 1). We based our estimate on a 90% completeness matrix of all UCE loci, which amounts to 428 loci and 199,275 aligned base pairs. We provided the above‐described ML phylogeny as input, used GTR as the substitution model and designated Panurginae and Oxaeinae as outgroups.

**TABLE 1:** Secondary calibrations used for the divergence time estimate analyses with RelTime (Tamura et al. 2012).

Calibrated node	calibrated mean	Calibrated σ	Distribution	Resulting 95% CI	Node age in Bossert et al. (2022); mean (95% highest posterior density)
Crown age of Andreninae	58.5	5	Normal	48.70–68.30	58.0 (48.79–69.18)
Divergence of *Ancylandrena* and *Megandrena*	34.85	4.35	Normal	26.32–43.38	34.85 (26.46–43.80)
Divergence of *Callandrena* clade 1 with remaining subtending *Andrena*	21.55	2.6	Normal	16.45–26.65	21.55 (16.44–26.71)
*Plastandrena* crown age	7.12	1.5	Normal	4.18–10.06	7.13 (4.22–10.37)
Divergence of *Trachandrena*, *Scrapteropsis*, *Onagrandrena*, *Diandrena*	10.54	1.8	Normal	7.01–14.07	10.55 (7.16–14.34)
Crown age of *Andrena* s. str.	5.73	1.2	Normal	3.38–8.08	5.73 (3.86–8.10)
Divergence of *Andrena* s. str. + *Cnemidandrena*	9.22	1.6	Normal	6.08–12.36	9.23 (6.13–12.99)
Divergence of *Ptilandrena* + *Euandrena*	7.86	1.5	Normal	4.92–10.8	7.86 (5.14–11.17)

*Note:* Ages are in millions of years.

### 2.6 Comparison of COI Barcode Data

We examined DNA sequences of the cytochrome c oxidase subunit I (hereafter ‘COI’) of *Andrena androfovea* n. sp. from both the Texas and the Oklahoma populations. This gene region is often referred to as the ‘COI barcode’ and is widely used for species recognition and identification (Hebert et al. 2003). After gaining an initial insight into the phylogeny of the new species based on the above‐described UCE phylogenomics, we determined a close relationship to *Andrena* s. str., which is why we obtained and used a COI barcode reference of a common member of this subgenus. To this end, we used the Phyluce script phyluce_assembly_match_contigs_to_barcodes and provided the COI barcode of *Andrena milwaukeensis* as reference (sample ID BEECC462‐08 from boldsystems.org), which corresponds to the traditional COI barcode used in the Barcode of Life Data System (Ratnasingham and Hebert 2007). From the returned matches, we used the two extracts with the higher sequencing depth for comparison.

### 2.7 Terminology and Documentation

Descriptive terminology follows LaBerge (1967) with the use of the standard abbreviations T (for tergum), S (for sternum), and F (for flagellar segment). Lengths of morphological structures are measured in mm. Values are presented as ± standard deviation. In addition, we add the term ‘distifemoral plate’ for the plate‐like structure structurally similar to the bastibial plate, which is found on the outer anterior margin of the hind femur adjacent to the bastibial plate. It is found in all *Andrena* species that we have examined and is best developed in females where it is present as a triangular‐shaped plate, mostly hidden by the tuft of hair at the apex of the hind femur. In male *Andrena*, the distifemoral plate is usually reduced to a small, flattened finger‐like projection. Among other andrenids we have examined, the distifemoral plate is present, albeit in somewhat modified form, in *Euherbstia*, *Megandrena*, and *Ancylandrena* (we have not examined *Orphana* and *Alocandrena*). The plate is present but reduced in *Nolanomelissa* and *Panurgus*, and is very reduced or absent in Calliopsini, Neffapini, Panurginini, Perditini, and Protandrenini. In contrast, it is large and conspicuous among female Oxaeinae where it is larger than the basitibial plate, although much reduced in males. Michener (1944) noted the presence of this structure in *Protoxaea* but did not name it or mention its presence in other bees. Hurd and Linsley (1976) described the plate in their characterization of the Oxaeinae (their Oxaeidae) but did not give it a name.

## 3 Results

### 3.1 Genome Sequencing

We sequenced whole‐genomic DNA of two representatives of *Andrena androfovea* n. sp. and retrieved 75,102,850 (WSUX_AV4) and 51,451,144 (WSUX0070) of short‐read Illumina reads, respectively. Assembling the short reads of the Oklahoma sample of *A. androfovea* (WSUX0070) with the de‐novo assembler SPAdes led to a genome assembly of 232.79 Mb length, 90,790 contigs, and an N50 value of 22,488. Average contig coverage, as inferred by SPAdes, is 27.55×. BUSCO v. 5.7.1 (Manni et al. 2021), which examines the presence or absence of 5991 single‐copy, orthologous genes, recovered the following values: 87.6% complete and single‐copy, 0.1% complete and duplicated, 8.4% fragmented, and 3.9% missing. Mining UCEs from this genome under the 80% identity and coverage parameters, we recovered 2489 UCEs, corresponding to 5,159,476 bp of UCE sequence data, with an average contig length of 2113.1 bp and 2484 contigs having over 1 kb length. For the sample from Texas, assembly statistics are as follows: 259.02 Mb length, 11.37× coverage, 261,725 contigs, and N50 of 2458. The BUSCO assessment led to the following recovery: 55.1% complete and single‐copy, 0.1% complete and duplicated, 23.5% fragmented, and 21.3% missing. A total of 2362 individual UCE loci were recovered from this genome, corresponding to 3,716,121 bp of UCE sequences with an average contig length of 1573.3 bp, and 1965 contigs being longer than 1 kb.

### 3.2 Phylogeny and Antiquity of *Andrena Androfovea*

Combining various genomic sequence data of Andreninae using the UCE Phyluce pipeline led to an 80% completeness matrix of 264 samples and 261 species (249 species of *Andrena*), comprising 1175 concatenated individual UCE loci and 555,734 bp. The average UCE length of the individual alignments was 472.97 bp, with an average of 14.5% missing data.

**FIGURE 1:**
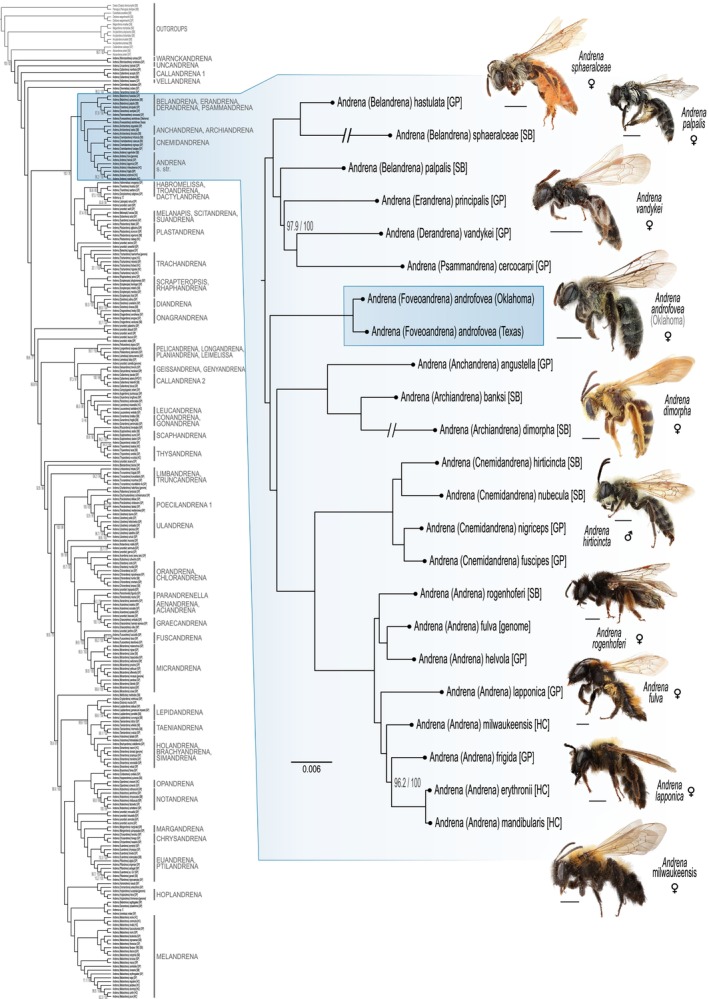
Phylogeny of *Andrena*, with the clade including the new subgenus *Foveoandrena* n. subg. enlarged. The phylogeny is based on maximum likelihood analysis of 1175 individual UCE loci. Support values are SH‐like approximate likelihood ratio tests and ultrafast bootstrap approximations and (SH‐aLRT/UFBoot2). Left scale bar indicates substitutions per site and scale bars next to specimen photos show 2 mm length. Initials in brackets after taxon names indicate the source of the sample: GP—Pisanty, Richter, et al. (2022), HC—Grab et al. (2019), SB—Bossert et al. (2022), and genome identifiers are listed in the methods section. The phylogeny is available as Nexus tree file in the FigShare archive associated with this article (https://doi.org/10.6084/m9.figshare.26352121).

Reconstructing phylogeny using these data in a maximum likelihood framework recovered the relationships shown in Figure 1 as being most likely. The two representatives of *A. androfovea* n. sp. form a distinct lineage which is sister group to a clade comprising the subgenera *Anchandrena* (two species, Western Nearctic), *Archiandrena* (three species, Eastern and Central Nearctic), *Cnemidandrena* (52 species, Holarctic), and *Andrena* s. str. (~80 species, Holarctic) with the following relationship: *Andrena androfovea* ((*Anchandrena* + *Archiandrena*) + (*Cnemidandrena* + *Andrena* s. str.)). Confidence for this phylogenetic relationship on the subgenus level is very strong with greatest possible support for both bootstrap and SH‐aLRT support values (Figure 1). The newly sequenced sample named *Andrena* sp. ‘L’ was recovered as a close relative to *Andrena violae* Robertson, and *Andrena* sp. ‘C’ is likely related to the recently described *Andrena nahua*. These two samples likely represent undescribed species of the genus *Andrena*, however, additional morphological study is necessary to confidently establish this.

Comparing the two extracted sequences of the DNA barcode region of the COI gene, we found near‐identical sequence data for the examined specimens from Texas and Oklahoma. Specifically, over a stretch of 656 base pairs, only three positions differed between the samples, corresponding to a pairwise distance of approx. 0.46%.

**FIGURE 2:**
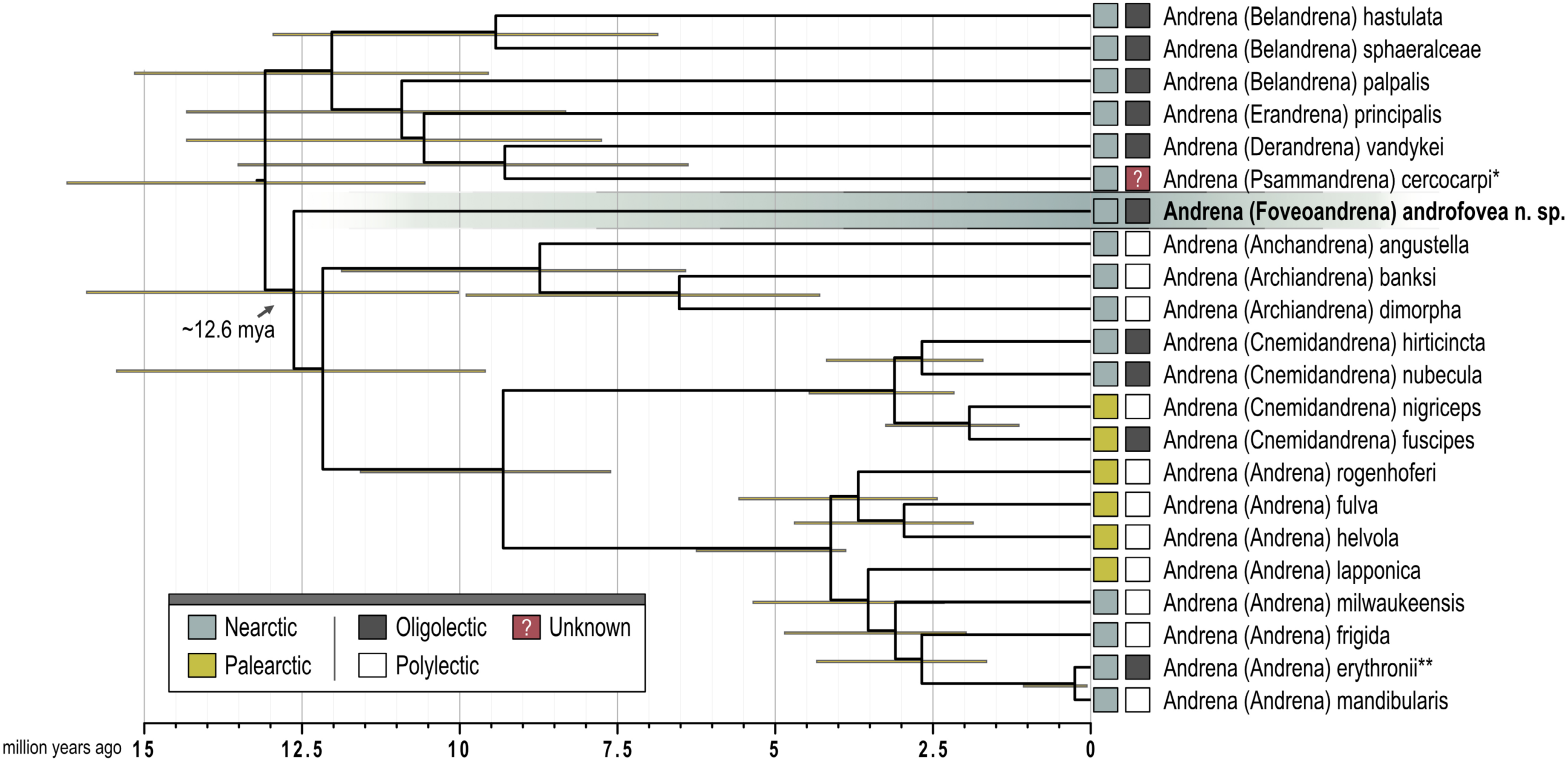
Divergence time estimates of *Foveoandrena* n. subg. and closely related subgenera of *Andrena*. Node bars show 95% confidence intervals of the divergence estimates. *Andrena cercocarpi*, indicated by a single asterisk, may be a specialist on *Eschscholzia* (Papaveraceae). *Andrena erythronii*, marked with two asterisks, is likely oligolectic on *Erythronium* (Liliaceae), but further study is needed.

According to our divergence time estimates, the newly described *Andrena* subgenus *Foveoandrena* likely diverged from the most recent common ancestor (MRCA) with its sister clade, which comprises *Anchandrena*, *Archiandrena*, *Cnemidandrena*, and *Andrena* s. str., around 12.6 million years ago (mya; 95% confidence interval 10.02–15.91 mya; Figure 2). The sister group comprises both Palearctic and Nearctic species; however, all other *Andrena* that we found as sister group to *Foveoandrena* plus its sister group are Nearctic species. Similarly, the sister group to *Foveoandrena* includes a mix of polylectic and oligolectic species, while the sister group to both *Foveoandrena* and its sister group seems to include exclusively oligolectic species, albeit on a range of distantly related host plants.

### 3.3 Systematics

#### *Andrena* Fabricius

##### *Foveoandrena* n. subg.

Type Species: *Andrena androfovea* Neff, Bossert and Hung, described below.

**FIGURE 3:**
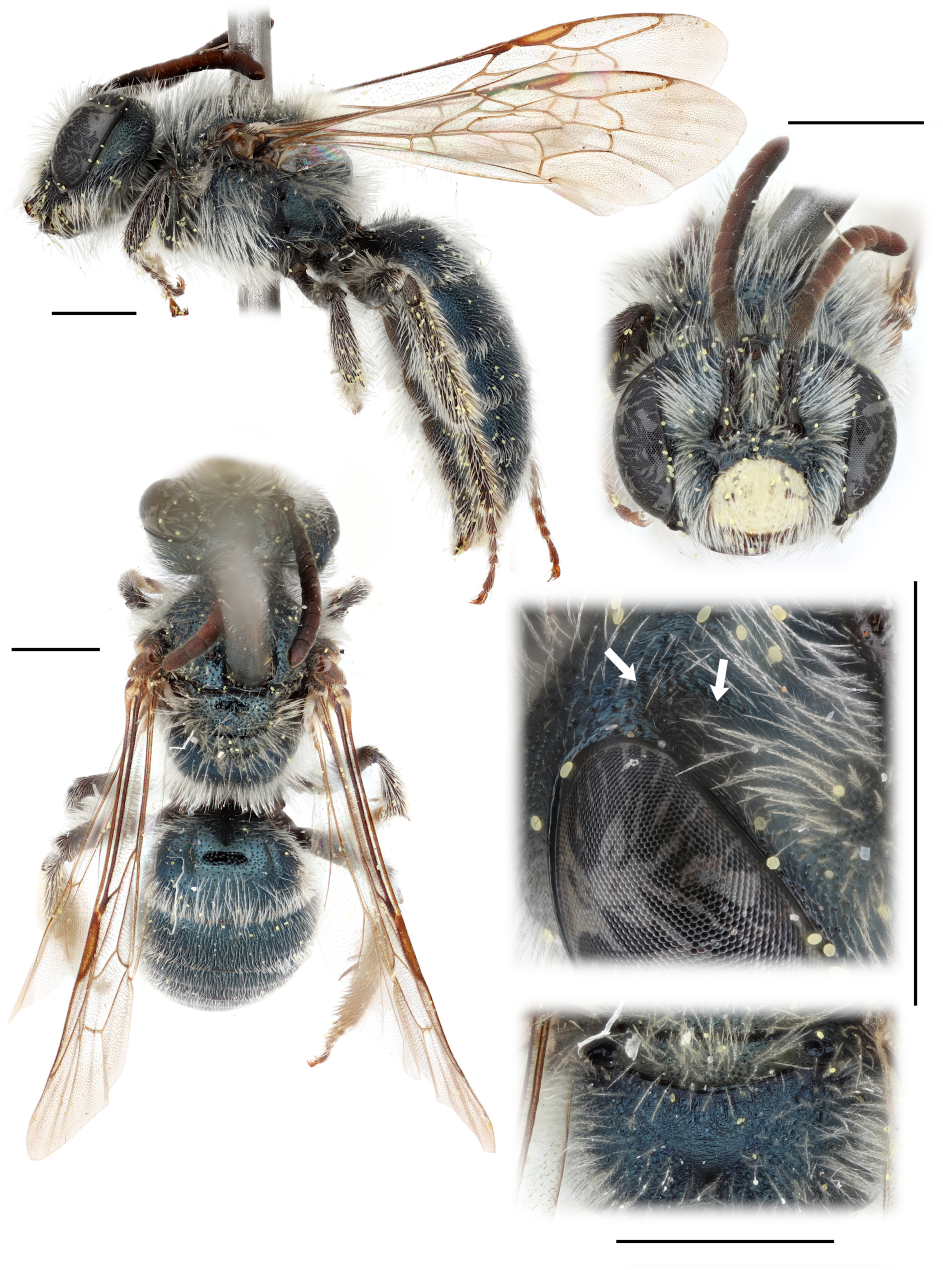
Male paratype of *Andrena* (*Foveoandrena*) *androfovea* n. sp. and n. subg. White arrows point to the characteristic fovea. Scale bars show 1 mm.

This monotypic subgenus is known from the southwestern margins of the Great Plains in western Texas and central to western Oklahoma. It is distinguished by its metallic coloration, the coarse punctation of both sexes, and the unique combination of the following sex‐specific characters: the males have small but distinct facial foveae (Figure 3), short female‐like mandibles, and a pygidial plate indicated by a narrow raised triangular area on T6, while the females have a reduced subgenal coronet and distinct sternal brushes of erect hair.

**FIGURE 4:**
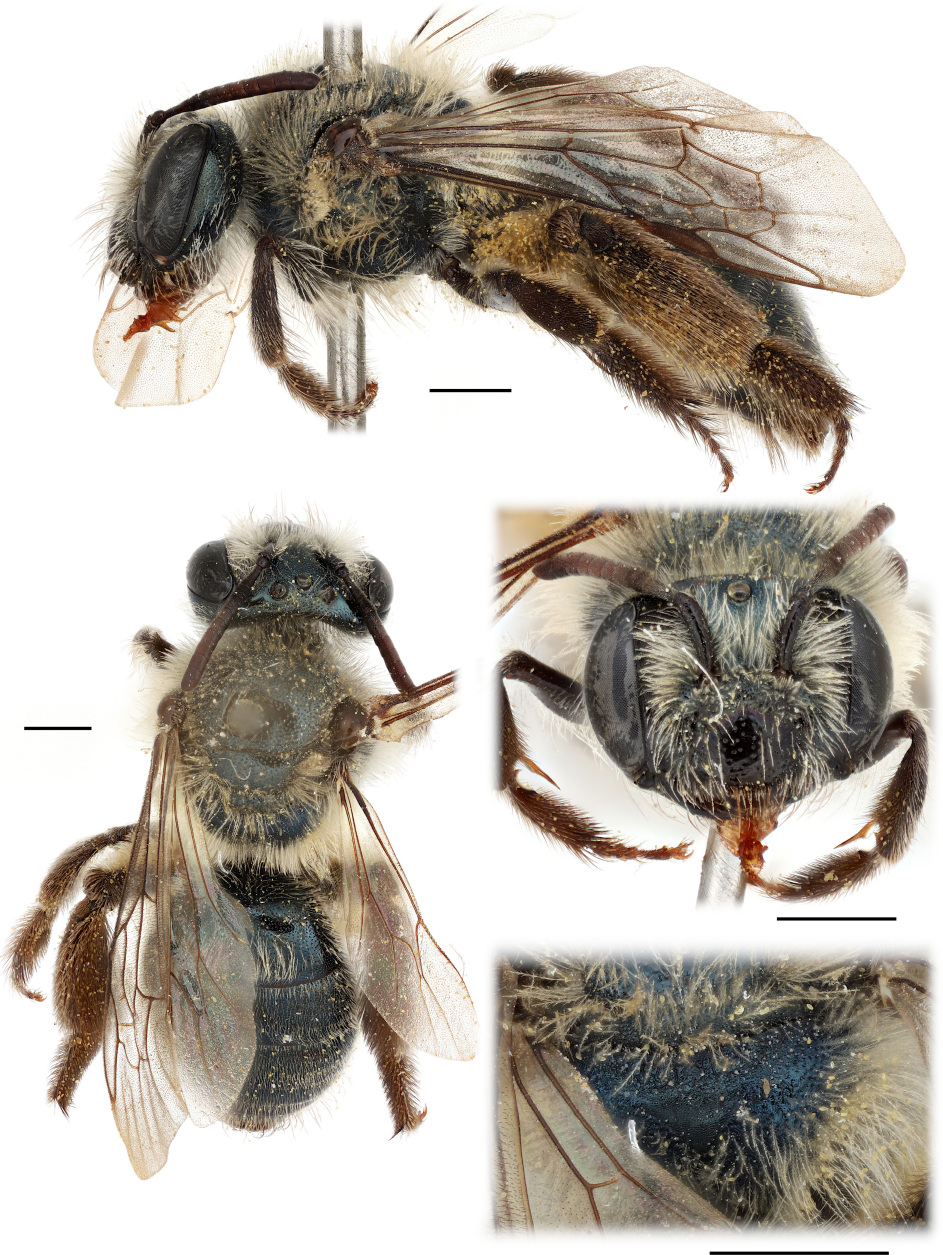
Female paratype of *Andrena* (*Foveoandrena*) *androfovea* n. sp. and n. subg. Scale bars show 1 mm.

Common characters: Small bees; integument mainly metallic dark blue to blue‐green (Figures 3 and 4). Facial quadrangle slightly broader than long; eyes with inner margins nearly parallel, slightly diverging above; clypeus punctate with interspaces shiny, vertex above lateral ocellus less than one ocellar diameter; genal area nearly as broad as eye; galea narrowed distally to broad triangle, apex narrowly rounded; maxillary palps longer than galea; labial palps shorter than galea; labral process weakly emarginate; mandibular basoventral lamella absent. Pronotum without humeral angle or dorsoventral ridge. Metapostnotum finely sculptured, basal area weakly rugulose. Pterostigma broad, three submarginal cells, first recurrent vein meets second submarginal cell in posterior third or more. Tibial scopal hair simple, hind tibial spurs normal; tergal apical fascia very weak, broadly interrupted medially.

**FIGURE 5:**
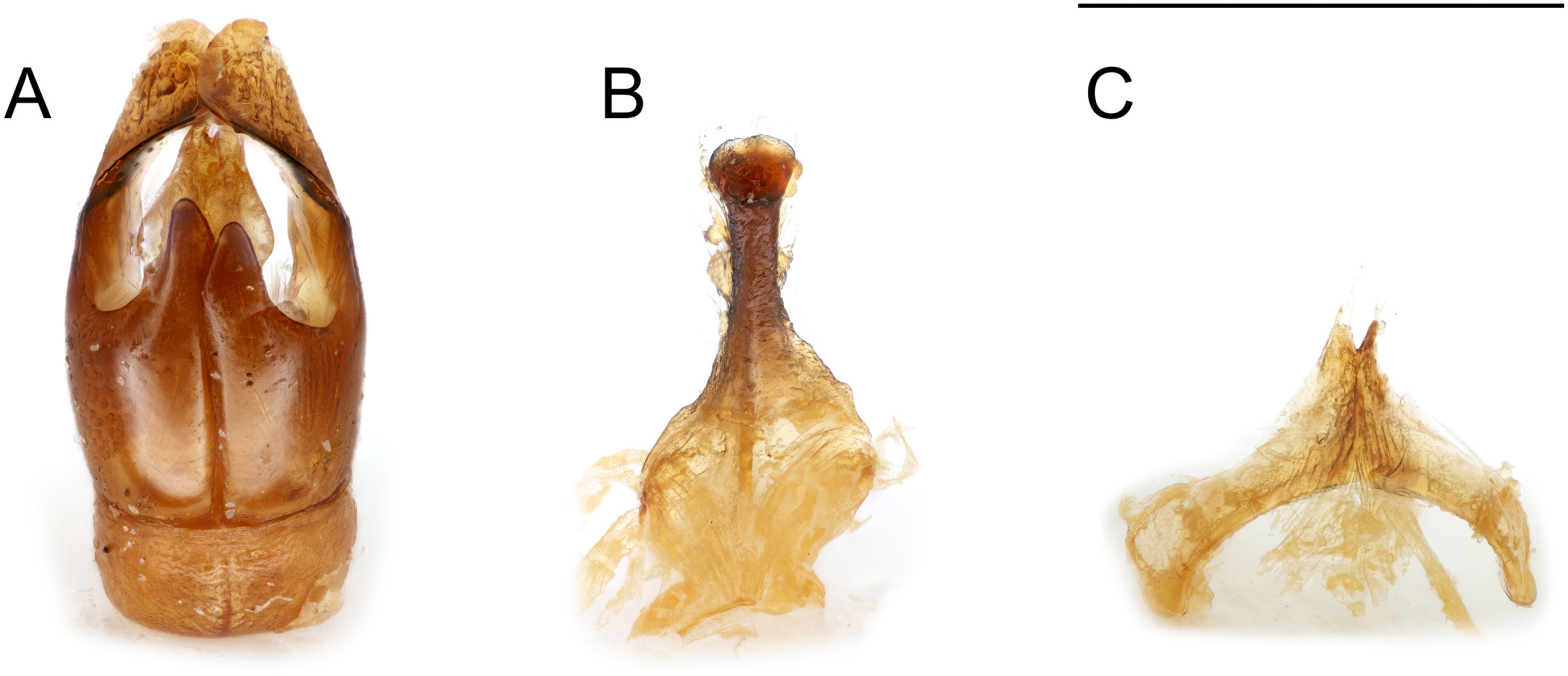
Genital capsule and hidden sterna of male *Andrena* (*Foveoandrena*) *androfovea* n. sp. and n. subg. (A) Genital capsule, dorsal view. (B) Sternum 8, ventral view. (C) Sternum 7, ventral view. Scale bars show 1 mm.

Male (Figure 3): Clypeus yellow. Facial fovea small but distinct. Mandibles short, female‐like, without distinct subapical tooth; antenna moderately long, F1 longer than wide, length F1 equals combined lengths F2 + F3; S6 flat, apex weakly emarginate. Pygidial plate indicated by raised, smooth, bare area on T6. Terminalia (Figure 5) without unique subgeneric characters.

Female (Figure 4): Facial fovea shallow, broad above extending from summit of eye almost to lateral ocellus but gradually narrowing below, occupying approximately half of space between eye and antennal insertion at lower margin; malar space linear; subgenal coronet reduced to row of short, branched hair; flagellum with F1 elongate, longer than F2 + F3. Propodeal corbicula incomplete, i.e., anterior hair row absent, interior with long erect hair throughout; tibial scopa of dense, long, simple hair; S2‐4 with apical fascia, discs of S2‐5 with brushes of erect simple hair. Pygidial plate triangular, apex rounded, without raised median area.

Etymology: The name refers to the presence of obvious facial foveae in the male, a rare character among North American *Andrena*.

##### *Andrena* (Foveoandrena) *androfovea* n. sp. Neff, Bossert and Hung

Diagnosis: Both sexes of *Andrena androfovea* can be distinguished from other metallic blue North American *Andrena* by the coarsely punctate scutum and metasomal terga, three submarginal cells, as well as the males possessing small but distinct facial foveae, short mandibles, and a reduced pygidial plate while the females have distinct sternal brushes of erect hair.

###### Male

Measurements (*n* = 5; in mm): body length = 7.1 ± 1.0 (5.6–8.6); head width = 2.5 ± 0.1 (2.2–2.6); wing length = 5.8 ± 0.5 (5.9–6.4); mesosomal width = 2.4 ± 0.2 (2.1–2.6); metasomal width = 2.5 ± 0.2 (2.1–2.6).

Color: Head metallic blue (with greenish hint in the Oklahoman specimens) except clypeus cream with apex, lateral extensions, and usual medial spots black; labrum, mandibles, scape, pedicel, and F1 and F2 dark brown to black; distal flagellar segments reddish brown; surface of fovea gray‐black. Mesosoma metallic blue except tegulae translucent light brown, wings clear with veins dark brown and stigma translucent light brown; legs brown to black; strigilus pale tan, tibial spurs dark brown with tips colorless. Metasoma with terga metallic blue, sterna black with weak metallic hints.

Structure: Head broad, 1.36 × as wide as long; facial quadrangle with width = 1.05 × length, eye length = 0.76 × head length; genal width = 0.87 × lateral eye width; clypeal length = 0.39 × head length; eyes slightly divergent above, upper inter‐ocular distance = 1.11 × lower inter‐ocular distance with upper inner margins slightly incurved at summit; ocello‐occipital distance narrow, 1.1 × ocellar diameter; ocello‐ocular distance = 3.1 × ocellar diameter. Facial fovea oval, small but distinct, extending along eye from just above summit of eye to 2/3 of distance to level of antennal insertion, broadest above occupying approximately 40% of space between lateral ocellus and eye margin, narrowing below, surface not obviously depressed but dull and micropunctate, contrasting with the metallic, striate, more coarsely punctate adjacent area of frons. Clypeus slightly produced, surface of disc smooth and shiny, with strong punctures 1–2 puncture widths apart, sparser on median third, becoming slightly smaller and denser on lateral margins. Parocular area finely, densely punctate, on shiny ground, punctures approx. 0.5–1 puncture widths apart; supra‐clypeal area densely punctate grading into more sparsely punctate area on frons below median ocellus, this with short median carina reaching to half distance to ocellus; frons striato‐punctate adjacent to fovea. Vertex with small area behind summits of eyes shiny and sparsely punctate, otherwise dulled by continuation of sculpture of frons; genal area with surface smooth, shiny, sparsely punctate near eye with puncture density increasing posteriorly; postgenal area densely punctate, surface dulled by fine tessellation. Antenna moderate in length, slightly extending past tegulae in repose, scape = 0.31 × head length, F1 = 1.4 × as long as its apical width, F2 and F3 distinctly shorter than wide with combined lengths = length F1. Malar space linear. Mandibles short, female‐like, without distinct subapical tooth, mandible length = 0.7 × intermandibular distance. Labral process short, trapezoidal, length = 0.33 × basal width, apical margin = 0.6 × basal width, apical corners rounded and apical margin weakly emarginate, surface dulled by fine transverse striae. Galea short, length = 0.25 × head length, surface weakly dulled by fine tessellation, apex broadly triangular. Glossa short, 0.15 × head length, stipes = 0.38 × head length, surface dulled by fine striation. Maxillary palps with combined length = 1.45 × galeal length, ratio of segments 10:6:7:5:5:4; labial palps with combined length = 0.62 × galeal length, ratio of segments 6:4:3:2.

Mesosoma: Pronotum evenly rounded, humeral angle and lateral ridge absent, surface shiny with fine, dense punctation; scutum smooth, shiny with strong punctures approx. 0.5–1 puncture widths apart on anterior half becoming sparser postero‐medially; scutellum similar but very sparsely punctate medially; metanotum finely punctate with surface dulled by fine tessellation; metapostnotum weakly rugulose basally, otherwise finely tessellate; lateral area of propodeum shinier, finely tesselate with fine punctures 1.5–2 puncture widths apart. Mesepisternum ruguloso‐punctate, surface dull; metepisternum impunctate, dulled by fine tessellation. Tegulae densely punctate on anterior fourth, remainder smooth and shiny. Wings with three submarginal cells, 2nd submarginal short with length = 0.61 × length 3rd submarginal on posterior margin, 2nd submarginal receiving first recurrent vein in posterior third. Legs with surfaces shiny, densely punctate with punctures mirroring hair patterns. Width hind tibia = 0.19 × length hind tibia, width hind basitarsus = 0.55 × width hind tibia and = 0.19 × length hind basitarsus. Tibial spurs normal, unmodified, posterior margins microserrate. Distifemoral plate small, triangular. Basitibial plate moderate, ovoid, length = 0.19 × tibial length, surface shiny, with fine dense puncture basally becoming larger and sparser distally. Tarsal claw with basal teeth well developed.

Metasoma: T1 with dorsal surface shiny with strong punctures approx. 1 puncture width apart distally, becoming slightly sparser on anterior dorsal surface, anterior face smooth, shiny, and impunctate. T2–4 similar but separation of punctures similar throughout; T5 similar but surface dulled by fine tessellation; surface T6 obscured laterally by dense hair; pygidial plate indicated by an apico‐medial, smooth, bare, raised apical area, not well defined laterally. Sterna dull with coarse punctation approx. 1 puncture width apart, sternal apices depressed, narrowly shiny, and finely punctate. S6 with apex weakly emarginate, not reflexed. S7 with median process short, deeply emarginate (Figure 5C). S8 with distal process entire (Figure 5B). Gonostylus relatively narrow basally, flattened, and expanded distally with rounded distal margin; penis valves expanded basally (Figure 5A).

Vestiture: Hair entirely white except with yellow‐orange hints on hair of inner surfaces of tarsi. Face with hair relatively dense, erect, and finely branched but nowhere obscuring surface; clypeal apical margin with conspicuous fringe. Hair sparser on vertex and genal area but dense in hypostomal area. Scape with conspicuous erect hair. Fovea with sparse, very short semi‐erect hair, not obscuring surface, becoming slightly longer below. Mesosoma with hair similar to that of head, metapostnotum bare.

Hair of legs typical for a male *Andrena*, with usual basitarsal brushes on inner surfaces. Distifemoral brush reduced. Basitibial plate with sparse hair (but with short appressed hair in one male from Oklahoma). Metasoma: T1‐5 very sparsely hairy, discs of terga with short erect hair obscuring surface, densest laterally; T2‐4 with dense apico‐lateral patches of long appressed hairs and very weak apical fascia, apical fascia stronger on T4 and T5 with apico‐lateral patch absent on T5; T6 and T7 with long appressed hair obscuring surface (except pygidial area of T7). Sterna 2‐5 with strong apical fascia of erect, distally angled hair; discs of all sterna with similar but sparser arrays of erect, slanted hair.

###### Female

Measurements (*n* = 10; in mm): body length = 8.3 ± 0.4 (7.3–8.9); head width 2.7 ± 0.1 (2.5–2.8); wing length 5.8 ± 0.3 (5.2–6.7); mesosomal width 2.7 ± 0.1 (2.6–2.8); metasomal width 2.9 ± 0.1 (2.8–3.1).

Color: Head and mesosoma as in male except clypeus dark brown to black. Metasoma as in male except T5 metallic blue basally with posterior half black and pygidial plate black.

Structure: Head broad, 1.37 × as wide as long; facial quadrangle with width = 1.05 × length; eye length = 0.7 × head length; genal width = 0.77 × lateral eye width; clypeal length = 0.38 × head length; eyes subparallel, slightly diverging above with upper inter‐ocular distance = 1.05 × lower inter‐ocular distance, although slightly incurved at summit; ocello‐occipital distance narrow, 0.8 × ocellar diameter; ocello‐ocular distance = 2.1 × ocellar diameter. Fovea broad above, extending from summit of eye almost to lateral ocellus but gradually narrowing below, occupying approx. half of space between eye and antennal insertion at lower margin. Clypeus slightly produced, surface of disc smooth and shiny, sparsely punctate with strong punctures 2–3 puncture widths apart, punctures slightly smaller and denser on margins. Parocular area finely, densely punctate, on shiny ground, punctures approx. 1 puncture width apart; supra‐clypeal area densely punctate grading into more sparsely punctate area on frons below median ocellus, this with short median carina reaching to half distance to ocellus; frons striato‐punctate adjacent to fovea. Vertex shiny, impunctate between eye and lateral ocelli but median area densely punctate with V‐shaped depression behind medial ocellus; genal area shiny with dense punctures, approx. 0.5–1.0 puncture widths apart; postgenal area obscurely punctate, surface dulled by fine tessellation. Antenna with scape = 0.31 × head length, F1 = 2.1 × as long as its apical width, F2 and F3 distinctly shorter than wide with combined lengths = 0.71 × length F1. Malar space linear. Mandible without distinct subapical tooth, mandible length = 0.6 × intermandibular distance, basoventral flange absent. Subgenal coronet reduced, indicated by row of short, branched hairs. Labral process short, trapezoidal, length = 0.33 × basal width, apical margin = 0.6 × basal width, apical corners rounded and apex weakly emarginate, surface dulled by fine transverse striae. Galea short, length = 0.26 × head length, surface weakly dulled by fine tessellation, apex broadly triangular. Glossa short, = 0.16 × head length, stipes = 0.38 × head length, surface dulled by fine striation. Maxillary palps with combined length = 1.20 × galeal length, ratio of segments 5:8:6:4:3:3. Labial palps with combined length = 0.58 × galeal length, ratio of segments 5:4:3:2.

Mesosoma: Pronotum evenly rounded, humeral angle and lateral ridge absent, surface shiny with fine, dense punctation; scutum smooth, shiny with strong punctures approx. 0.5–1 puncture widths apart on anterior half becoming sparser postero‐medially; scutellum similar but very sparsely punctate medially; metanotum finely punctate with surface dulled by fine tessellation; metapostnotum dull, anterior half with low, irregular, anastomosing rugulae and posterior half dull and impunctate; lateral area of propodeum shinier, finely tesselate with fine punctures 1.5–2 puncture widths apart. Mesepisternum ruguloso‐punctate, surface dull; metepisternum impunctate, dulled by fine tesselation. Tegulae densely punctate on anterior fourth, remainder smooth and shiny. Wings with three submarginal cells, 2nd submarginal short with length = 0.61 × length 3rd submarginal on posterior margin; 2nd submarginal receiving 2nd recurrent vein in posterior third of cell. Legs with surfaces shiny, punctures mirroring hair patterns. Width hind tibia = 0.32 × length hind tibia, width hind basitarsus = 0.57 × width hind tibia. Tibial spurs normal, unmodified, posterior margins microserrate. Distifemoral plate large, subtriangular. Basitibial plate large, reniform, length = 0.29 × tibial length. Tarsal claw with basal teeth well developed.

Metasoma: T1 with dorsal surface shiny with strong punctures approx. 1 puncture width apart distally, becoming slightly sparser on anterior dorsal surface, anterior face smooth, shiny, and impunctate. T2–4 similar but separation of punctures similar throughout; T5 similar but surface dulled by fine tessellation; surface T6 obscured by dense hair. Pygidial plate large, subtriangular with broadly rounded apex, interior surface dull, margins impunctate but with fine, dense punctures medially (insertions of short erect setae). Sterna dull with coarse punctation approx. 1 puncture width apart mirroring hair patterns; sternal apices narrowly impunctate.

Vestiture: Head: Hair entirely pale except for few, long, simple, golden hairs arising from anterior clypeal fovea and mandibles with long, curved, black, simple hair. Hair mainly sparse, not obscuring surface except longer and denser in parocular area where it partially obscures surface. Fovea covered with short appressed white hair. Scape with long sparse pale hair with very short branches. Labral process bare, margin of labral process with row of erect, short simple hair; apical margin of labrum with dense row of very short simple hair. Stipital comb weak; margin of galea with short, sparse, curved yellow/tan hair. Mesosoma: hair pale, erect, and branched but not dense or obscuring surface. Propodeal corbicula incomplete, with anterior row of hair absent, interior with long, erect simple or weakly branched hair throughout, dorsal and posterior margins with dense rows of long, erect, finely branched hair.

Hair of foreleg entirely pale except dark brown on anterior (outer) surface of tibia and entirely dark on basitarsus. Hair of midleg similar. Hind leg with hair of coxa, trochanter, and femur entirely white except distifemoral brush dark (apparent color varies with angle of view); trochanteral flocculus of pale hair large, complete; scopal hair of hind tibia and basitarsus all dark, long, and simple except row of pale branched hair on posterior margin. Hair of inner surface of hind tibia and basitarsus dark. Basitibial plate completely covered with appressed short black hair, apparent color varies with angle of view. Metasoma: T1‐5 very sparsely hairy; T1 without apical fascia, disc with sparse, long, erect, simple, white hair, densest laterally; T2‐4 with very weak white apical fascia, broadly interrupted medially, apices with narrow, arched hairless areas, broadest medially, discs with sparse, very short, simple, erect white hairs; prepygidial fimbria of T5 well developed and black, disc of T5 with short, sparse, erect white hair; dense long hair of T6 black. Sterna 2–4 with apical fascia of erect, posteriorly slanting, white hair with very short branches; discs of sterna 2–5 with brushes of erect, fine, simple, white hair.

Variation: Some individuals, particularly those from the northern part of the species' range, are dark metallic green rather than blue.

###### Material Examined

Type material. Holotype: ♂, USA, Texas, Val Verde Co., Devils River, Dolan Falls Preserve, TNC, (28.713° N, 100.396° W), 9–11.III.2003, leg. A. Hook and J. L. Neff, 39908. Allotype: ♀, 39911, same locale but, 23–25.III.1995, leg. A.W. Hook. Paratypes: Oklahoma, Greer Co., 2 ♂♂, OKBS.POL.3596, OKBS.POL3595, Doc Hollis Lake, vicin, 34.989° N, 99.709° W, 30.V.2022, leg. K.L.J. Hung, ex flowers of *Quincula lobata*; 2 ♀♀, OKBS.POL.3628, OKBS.POL.3629, same data except ex flowers of *Chamaesaracha coniodes*; Custer Co., 3 ♀♀, OKSB.POL.8407, OKSB.POL.8408, OKSB.POL.8409, Washita N.W.R. (nr.), 35.595° N 99.278° W, 30.V.2022 30.V.2023, leg. S. O'Dell and K.L.J. Hung, ex flowers of *Quincula lobata*; Custer Co., 1 ♂, OKSB.POL.8410, Washita N.W.R. (nr.), 35.595° N 99.278° W, 30.V.2022 30.V.2023, leg. S. O'Dell and K.L.J. Hung, ex flowers of *Quincula lobata*. Texas: Bailey Co., 1 ♀, USGS‐DRO 520944 (Muleshoe National Wildlife Refuge, 20 mi. S), 33.953° N, 102.780° W, 25.IV.2016, leg S. Droege 15273; Bexar Co., 1 ♀, USGS‐DRO 406304, (Medina River Greenway), 29.247° N, 98.532° W, 15.III,2016, leg S. Droege 14069; Dimmit Co., 2 ♀♀, 26330, 26331. Chaparral W.M.A., 29.332° N, 99.414° W, 14‐16.III.1990, leg. A.W.Hook; Maverick Co., 2 ♀♀, 26330, 26331; Eagle Pass, 10 mi. NE, 28.781° N, 100.390° W, 5.IV.2004, leg. J.L. Neff, both ex flowers of *Nerisyrenia camporum*; 1♀, K0782. Eagle Pass, 18 mi. NE, 28.892° N, 100.248° W, 17.III.1990, leg. J.L. Neff, ex flowers of *Physaria recurvata*; 4 ♀♀, 09825, 09826, 09827, 09828, Eagle Pass, 1 mi. E, 28.710° N, 100.457° W, 5.IV.2004, leg. J.L. Neff, all ex flowers of *Quincula lobata*; 1 ♀, 26351, same locale as preceding but 5.III.2004, ex flowers of *Chamaesaracha* sp.; 1 ♂, 28243, El Indio, 8.5 mi. S., 28.391° N, 100.293° W, 11.III.2005, leg. J. L. Neff, ex flowers of *Physaria lasiocarpa*; Taylor Co., 1 ♀, X132976, Dyess Air Force Base, 32.441° N, 99.111° W, 12‐13.IV.2001, leg. H.W. Ikerd, ex yellow pan trap; Val Verde Co., 4 ♂♂, same data as holotype (39,909, 39,910, 39,912, 39,914), 2 ♀♀, Del Rio, 7 mi. N. W., 29.093° N, 99.956° W, 21.III.2003, leg. J. L. Neff, ex flowers of *Chamaesaracha* sp., Webb Co., 2 ♀♀, K04600, K04601, Laredo, 3 mi. SE, 27.421° N, 99.482° W, 21.III.1987, ex flowers of *Quincula lobata*, 1 ♀, K04603, same data except ex flowers of *Chamaesaracha sordida*.

The holotype and paratype will be deposited at USNM. Paratypes will be deposited at TAMUIC, UTIC, SEMC, OMNH, WSUC and the personal collections of the authors.

Etymology: The name refers to the presence of distinct male facial foveae, a rare occurrence in North American *Andrena*.

## 4 Discussion

### 4.1 Phylogeny and Antiquity of *Andrena* and *Foveoandrena*

The presented phylogeny of *Andrena* is the most species‐rich molecular‐based phylogeny of the genus to date, except for COI barcode trees. We combined the sequence data from the groundbreaking *Andrena* study of Pisanty, Richter, et al. (2022) with the first comprehensive phylogenomic study of Andrenidae (Bossert et al. 2022) and developed a taxon‐rich framework for the second‐largest genus of bees in the world. With 249 included species of *Andrena*, we present phylogenetic relationships for about 14% of the described species of the genus. Optimizing the assembly recovery improved the matrix size and density over the previous work that aimed primarily at *Andrena*: we recovered 1175 UCE loci with 80% taxon completeness, totaling 555,734 aligned nucleotides. This compares favorably to the results of the study that provided the majority of the samples (Pisanty, Richter, et al. 2022), which had 1009 loci at 75% taxon completeness, yielding 419,858 aligned nucleotides.

Reciprocally accounting for the lack of included lineages, our topology of *Andrena* phylogeny is highly similar to those of the two previous studies that included significant representatives of Andreninae (Bossert et al. 2022; Pisanty, Richter, et al. 2022). The phylogenies among subgenera of *Andrena*, including the early branching patterns, are largely congruent. A notable exception is the placement of *Cubiandrena*. *Cubiandrena* was considered a subgenus of *Andrena*, until Pisanty, Richter, et al. (2022) recovered it as the sole sister genus to *Andrena*. Given its striking morphology, they formally reinstated *Cubiandrena* as a genus (as initially proposed by Dubitzky, Plant, and Schönitzer (2010)). We here recovered *Cubiandrena* as sister group to the South American endemic and monotypic genus *Alocandrena*. *Alocandrena* behaved like a rogue taxon in the recent studies on *Andrena*: Pisanty, Richter, et al. (2022) found it as sister group to *Ancylandrena* + *Megandrena*, Bossert et al. (2022) as sister group to *Andrena* + *Cubiandrena*, and Ramos, Martins, and Melo (2022) as sister group to *Andrena* (albeit without having sampled *Cubiandrena*). A sister group relationship of *Alocandrena* and *Cubiandrena* has not been previously reported and presents a surprising alternative phylogenetic placement. The distribution of these genera is extremely disjunct, with *Cubiandrena* being an East Mediterranean‐endemic genus of three species, one of which extends into northwestern Iran (Wood 2020), while *Alocandrena* is known only from a single species from the western slopes of the Andes mountains of central and northern Peru (Michener 1986; Rozen and Ugarte‐Peña 1999). However, the phylogenetic support for this placement is not as high as for the majority of our nodes: while most all nodes of the new phylogeny have the highest possible support of 100 SH‐like approximate likelihood ratio tests (SH‐aLRT) and ultrafast bootstrap approximations (UFBoot2), the node involving *Cubiandrena* and *Alocandrena* received only 98.7 and 82, respectively. Similarly, the node involving *Alocandrena* was not perfectly supported in the other studies with 97 Bootstrap support in Pisanty et al.'s (2022) preferred tree (their 232 T‐F75 matrix, rcluster) and 98 bootstrap approximations in Bossert et al. (2022). Fine‐scale sensitivity analyses of the early divergences of Andreninae, including denser taxon representation and greater quantity of genetic information will be needed to conclusively establish the patterns of early andrenine evolution.

Integrating the newly generated whole‐genome data into the new phylogenomic framework of *Andrena*, we found a well‐supported phylogenetic placement for the new species *Andrena androfovea*. We found the new species as sister group to a clade comprising several well‐established subgenera of *Andrena*, including *Andrena* s. str. and *Cnemidandrena* (Figure 1). If again accounting for the lack of included lineages in the respective studies (Bossert et al. 2022; Pisanty, Richter, et al. 2022), the phylogeny of the *Andrena* subgenera closely related to *Andrena androfovea* (i.e., the blue inset shown in Figure 1) is robustly supported here and entirely congruent with the phylogenies of Pisanty, Richter, et al. (2022) and Bossert et al. (2022). Interestingly, the phylogenetic placement of *Andrena androfovea* is comparatively isolated, as this single species represents the sister group to a clade that comprises at least 140 described species. This placement further necessitates the establishment and description of the new subgenus *Foveoandrena*, as formalized above. Problematic, however, is the status of the related subgenus *Belandrena*, and future research will need to address the taxonomy of this group. LaBerge (1985) considered *Belandrena* to comprise six species, united by males with strong humeral angles and both sexes with sharply pointed galeas. Our results show that *Belandrena* is very likely an artificial grouping as the four included species comprise three separate clades. Given that we have not sampled the type species of the subgenus, *Andrena nemophilae* Ribble, 1968, we cannot confidently state which clade may represent the true *Belandrena*. However, *A. nemophilae* is morphologically very similar to *A. sagittagalea* (Ribble 1968a), which we included, and which was found as distantly related to *Foveoandrena* and likely more closely allied to *Derandrena* (Figure 1). In this light, it appears that the ‘*Belandrena*’ that are more closely related to *Foveoandrena* will need to be delineated with new subgeneric concepts in the future.

According to our divergence time estimates, *Foveoandrena* diverged from the most recent common ancestor with its sister clade around 12.6 million years ago (Figure 2). This age is comparatively old for a lineage with only one known species to date. In comparison, we inferred a crown age of ~4.1 mya (stem age: 9.3 mya) for *Andrena* s. str., a subgenus with currently ~80 recognized species. This clearly shows much greater species diversification rates for *Andrena* s. str. in comparison to *Foveoandrena*. To this end, it is possible that *Foveoandrena* may include more species that remain to be discovered and that the clade once had greater species diversity which went extinct.

The phylogenetic placement of *Foveoandrena* indicates a lineage origin in the New World (Figure 2). While the sister group to *Foveoandrena* comprises a mix of Old and New World *Andrena*, the MRCA of the *Foveoandrena* and its sister clade combined was almost certainly distributed in the New World, as the earlier‐branching *Belandrena*, *Erandrena*, *Derandrena* and *Psammandrena* are all exclusively Nearctic in distribution, and primarily species of Western and Southwestern North America. Further, *Anchandrena* and *Archiandrena*, which diverge earliest in the sister clade of *Foveoandrena*, are also both exclusively Nearctic lineages. While our study does not include a formal, model‐based analysis of historical biogeography of *Andrena*, we see no reason to suspect an origin of *Foveoandrena* outside of the Nearctic.

### 4.2 Biology and Pollen‐Collecting Behavior

Our observations and collection records indicate that *Andrena androfovea* is a univoltine vernal bee with an apparent strong preference for pollen of two closely related genera of the Solanaceae: Physalideae (*Quincula* and *Chamaesaracha*). Earlier anatomical work suggested that *Quincula* and *Chamaesaracha* are sister taxa (Barbro, 1996) but the molecular analysis of Särkinen et al. (2013) indicated that they are just closely related. *Chamaesaracha* consists of ten species which range from central Texas to eastern California and from southern Kansas to the southern Chihuahuan desert (Averett 1993), while *Quincula* is a monotypic genus with a similar range (Sullivan 1993). Both are prostrate, or near prostrate, herbaceous perennials with upward‐facing rotate flowers with longitudinally dehiscing anthers. Although not directly tested, the behavior of bees and other floral visitors on the flowers indicates that the flowers are nectariferous. While differing in a number of technical details, the most obvious difference between the two are the pale yellow‐cream corollas of *Chamaesaracha* and the blue corollas of *Quincula*. With favorable conditions, flowering can occur at any time through the year, but the main flowering is typically in the spring in the eastern portion of their ranges but later, during the summer or early fall, usually in association with monsoon rains, in the western part of their ranges. In Texas and Oklahoma, the flowers are visited by a mix of generalist and specialist bees, the latter including *Calliopsis callops* (Cockerell and Porter), *Colletes latitarsis* Robertson, *C. scopiventer* Swenk, *C. swenki* Stephen, and *C. texanus* Cresson as well as *Perdita beameri* Timberlake, *P. chamaesarachae* Cockerell, *P. deltophora* Cockerell, *P. lenis* Timberlake, *P. munita* Timberlake *and P. sexmaculata*, but we have observed no other *Andrena* besides *A. androfovea* on these flowers.

The use of Solanaceae as a pollen host is unusual for any *Andrena* and specialization on members of the Solanaceae by *A*. *androfovea* would be unique for the genus. In an analysis of the scopal pollen of nine females with significant scopal pollen loads (although none had what would usually be considered a full load), we found that the loads consisted entirely (or nearly entirely) of *Chamaesaracha* or *Quincula* pollen, including one female caught on *Physaria recurvata* (A. Gray) O'Kane and El‐Shebaz. Other females collected on plants other than *Quincula* or *Chamaesaracha* lacked scopal pollen. As a result, all available evidence, while still slim, points to dietary specialization on *Chamaesaracha* and *Quincula* as pollen hosts. As seen in Figure 2, the closest relatives of *A. androfovea* comprise a mix of polylectic and oligolectic bees (with most being polylectic), while the clade basal to *Foveoandrena* and its sister group consists of species variously oligolectic on a mix of plant taxa (Malvaceae, Hydrophyllaceae, Ericaceae and probably Papaveraceae) which are neither closely related to one another nor to the Solanaceae (Zuntini et al. 2024).

As noted earlier, the split of *Foveoandrena* from its sister group occurred approximately 12.6 mya. A recent analysis of the Solanaceae indicates that the Physalideae arose around 42 mya with the split of *Chamaesaracha* with the remaining physalidines at approx. 24 mya (Huang et al. 2023), hence *Chamaesaracha* (or a *Chamaesaracha*‐like ancestor) would have been present at the origin of *Foveoandrena*.

**FIGURE 6:**
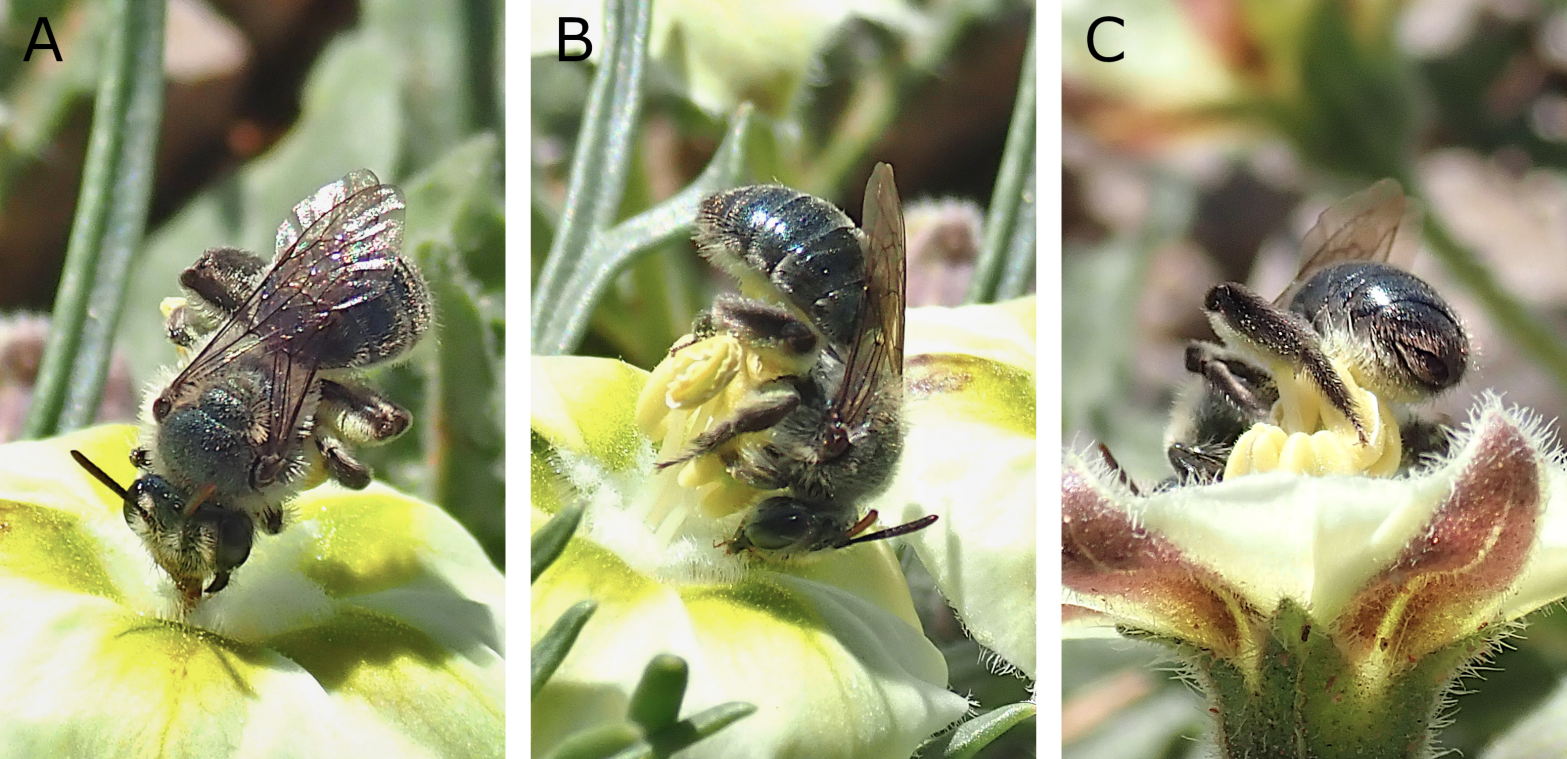
Flower visit of *Andrena* (*Foveoandrena*) *androfovea* n. sp. and n. subg. on *Chamaesaracha coniodes* (Moric. ex Dunal) Britton, showing nectar‐ and pollen‐collecting behavior. (A) Dorsal view (B) Lateral view. (C) Ventral view. Collection of yellow *C. coniodes* pollen on the sternal hairs can be seen in (C).

The dense brushes of erect hair on the metasomal sterna of female *A*. *androfovea* are similar to the brushes found on the venters of female *Colletes swenki* Stephen and *C. scopiventer* Swenk, both bees known to be vibratile pollen collectors on *Chamaesaracha* and *Quincula* (JLN, pers. obs.). However, careful field observations of *A*. *androfovea* foraging on *Ch*. *coniodes* (Moric. ex Dunal) Britton by one of us (KLJH) failed to detect any vibratile behavior. Rather, the females were observed to collect pollen from *Ch*. *coniodes* simultaneously while nectaring (Figure 6; video data in FigShare archiv, https://doi.org/10.6084/m9.figshare.26352121). A female would insert its mouthparts into a nectary while grasping the flower with its forelegs (Figure 6A). At the same time, it would scrape the laterally dehiscing anthers with the brushes on the inner surfaces of the basitarsi of its midlegs (Figure 6B). The hind legs were held motionless during these actions. Pollen was transferred to the hairs of the metasomal venter as well as the scopal hairs of the hind tibia during these brushing movements (Figure 6C). Later, pollen was presumably transferred to the propodeal corbicula and trochanteral flocculus for transport back to the nest. Judging from female specimens bearing varying amounts of pollen, the propodeal corbicula and trochanteral flocculus are loaded before finishing loading the tibial scopa.

### 4.3 Notes on Male Facial Foveae in *Andrena*

Well‐defined facial foveae covered with velvety hairs of female *Andrena* are a synapomorphy of that genus (Bossert et al. 2022; Pisanty, Richter, et al. 2022; Ramos, Martins, and Melo 2022). These foveae are underlain by glandular tissue and presumably play some role in chemical signaling during mating, although exactly what that is remains uncertain (Schuberth and Schönitzer 1993). While obvious in females, male *Andrena* typically lack obvious facial foveae and are commonly assumed to lack them entirely, an exception being *Andrena* (*Derandrena*) *timberlakei* Cockerell, whose males have large female‐like fovea with velvety hairs (Ribble 1968b). Although male facial foveae are usually difficult to detect, Schuberth and Schönitzer (1993) found that all males of the 86+ species of *Andrena* they examined possessed facial foveae, although in general they were smaller than those of their females, weakly differentiated from the surrounding cuticle, and lacking dense velvety hair covering the foveal surface. The foveae of male *Andrena androfovea* partially fit that description in that they are relatively small and lack a covering of velvety hairs, but differ in being well differentiated from the surrounding cuticle. Specifically, the cuticle is dull gray‐black in color and micropunctate, and contrasting with the metallic, striate, more coarsely punctate surface sculpturing of the adjacent area of the frons. It is well visible from a frontolateral angle (Figure 3).

## 5 Conclusion

With the description of *Andrena androfovea*, we introduce a previously unknown species and subgenus to science. By integrating multiple lines of evidence and documentation, from molecular phylogenomics to observations of bee and host plant interactions, we give an unusually detailed account on the biology and natural history of a previously unknown species, providing exemplary guidance for alpha‐taxonomic research on bees and other pollinating insects.

## Author Contributions

**Silas Bossert:** conceptualization (equal), data curation (equal), formal analysis (equal), investigation (equal), visualization (lead), writing – original draft (equal), writing – review and editing (equal). **Keng‐Lou James Hung:** conceptualization (equal), data curation (equal), formal analysis (equal), investigation (equal), writing – original draft (equal), writing – review and editing (equal). **John L. Neff:** conceptualization (equal), data curation (equal), formal analysis (equal), investigation (equal), writing – original draft (equal), writing – review and editing (equal).

## Conflicts of Interest

The authors declare no conflicts of interest.

## Data Availability

Raw illumina sequence reads are available in the Sequence Read Archive (SRA) under BioProject PRJNA1139224. Assembled contiguous sequences (contigs) of the two samples of *Andrena androfovea*, COI barcode sequences, final sequence alignments, as well as photo and video footage are available from the FigShare archive associated with this article (https://doi.org/10.6084/m9.figshare.26352121).
